# 

**Published:** 1914-10

**Authors:** 


### CONTENTS.

ORIGINAL COMMUNICATIONS—

A Trip Through Europe and the Hospitals, by L. C. Fischer, M. D. .............................289

A Study in Intestinal Peristalsis, by George M. Niles, M. D., Professor of Gastro-Enterology and Clinical Medicine, Atlanta Medical College ......304

Meeting of the Fulton County Medical Society, by R. R. Daly, M. D. ............................315

EDITORIAL ..........................................319

SELECTIONS AND ABSTRACTS ..................321

